# The Role of Single Nucleotide Polymorphisms in Predicting Prostate Cancer Risk and Therapeutic Decision Making

**DOI:** 10.1155/2014/627510

**Published:** 2014-02-19

**Authors:** Thomas Van den Broeck, Steven Joniau, Liesbeth Clinckemalie, Christine Helsen, Stefan Prekovic, Lien Spans, Lorenzo Tosco, Hendrik Van Poppel, Frank Claessens

**Affiliations:** ^1^Department of Urology, University Hospitals Leuven, Herestraat 49, 3000 Leuven, Belgium; ^2^Laboratory of Molecular Endocrinology, Department of Cellular and Molecular Medicine, KU Leuven, Campus Gasthuisberg O&N1, P.O. Box 901, Herestraat 49, 3000 Leuven, Belgium

## Abstract

Prostate cancer (PCa) is a major health care problem because of its high prevalence, health-related costs, and mortality. Epidemiological studies have suggested an important role of genetics in PCa development. Because of this, an increasing number of single nucleotide polymorphisms (SNPs) had been suggested to be implicated in the development and progression of PCa. While individual SNPs are only moderately associated with PCa risk, in combination, they have a stronger, dose-dependent association, currently explaining 30% of PCa familial risk. This review aims to give a brief overview of studies in which the possible role of genetic variants was investigated in clinical settings. We will highlight the major research questions in the translation of SNP identification into clinical practice.

## 1. Introduction

Prostate cancer (PCa) is a major health care problem because of its high prevalence, health-related costs, and mortality. Even though most patients have clinically localized and indolent tumors at diagnosis, worldwide, this disease still holds second place in the leading causes of cancer deaths [1]. Despite its prevalence, lethality, and socioeconomic burden, there are still many diagnostic and therapeutic challenges in the PCa field. This is mainly due to the lack of cancer- and/or patient-specific biomarkers, currently limiting patient-tailored diagnostics/therapeutics in PCa.

Age, race, and family history remain primary risk factors for the development of PCa. It has been shown that PCa is one of the most heritable cancers with epidemiological studies suggesting the role of genetics in PCa development [2, 3].

Due to the latter, there has been an increasing focus on the role of single nucleotide polymorphisms (SNPs) in the development and progression of PCa but also on their role in diagnostics and risk prediction. A SNP is a DNA sequence variation occurring when a single nucleotide (A, T, C, or G) in the genome differs from the normally expected nucleotide. These SNPs are known to underlie differences in our susceptibility to diseases. SNPs need to be determined only once and are easy to determine, making them interesting biomarkers. The rising interest in the role of SNPs in PCa development and progression is illustrated by the number of studies being published on SNPs in the PCa field.

In 2008, an extensive genome-wide association study (GWAS) compared SNPs between PCa cases and controls. Since then, numerous GWAS studies have been conducted [4–26]. While many SNPs were only moderately associated with PCa risk, in combination, they had a stronger, dose-dependent (i.e., cumulative effect of number of SNPs) association. A total of 77 susceptibility loci are currently explaining approximately 30% of the familial risk [6]. With ongoing GWAS, we could expect that more genetic variants will be found, explaining more of the PCa familial risk. However, the question has been raised whether finding more PCa risk-associated SNPs will have added value over the currently known ones [27].

Many SNPs are connected to each other through “linkage disequilibrium,” which is a nonrandom association of alleles at two or more loci, descendant from a single, ancestral chromosome. However, the SNPs detected through GWAS studies are mostly limited to “index SNPs,” excluding other SNPs which are in linkage disequilibrium. Clearly, these index SNPs are not necessarily the SNPs causative for its associated phenotype (i.e., PCa risk, risk of progression, etc). Therefore, molecular analyses will be needed to identify the exact SNP within each linkage domain which is the causative SNP. SNPs that lie within an open reading frame can lead to changes in messenger RNA stability or translation efficiency, as well as changes in structure/activity of the encoded proteins. However, most SNPs are located outside of the genes and are suspected to affect gene expression levels and genome/chromatin organization. Therefore, it is interesting to determine the role of these SNPs in the clinical field. This review aims to give a selected overview of studies on the possible role of genetic variants in clinical practice. We will highlight diagnostic and therapeutic obstacles which are currently major issues in clinical practice.

## 2. Evidence Synthesis

### 2.1. Early Detection

To detect PCa in its early stages, currently, clinicians are limited to serum PSA level measurements as a marker, which lacks sensitivity and specificity. Therefore, PSA screening (defined as mass screening of asymptomatic men) has been heavily debated. Two prospective studies (The Prostate, Lung, Colorectal, and Ovarian (PLCO) Cancer Screening Trial and The European Randomized Study of Screening for Prostate Cancer (ERSPC)) have contributed greatly to this discussion. The PLCO study concluded that PCa-related mortality did not significantly differ between patients being screened or not [3]. The ERSPC study inferred that, based on PSA-based PCa detection, 1410 men would need to be screened and 48 additional cases of PCa would need to be treated to prevent one death from PCa, resulting in a high rate of over diagnosis [28].

In reaction to these recent results, the US Preventive Services Task Force has radically recommended against PSA-based screening [29]. However, they ignored the 50% reduction in PCa-specific mortality since the introduction of PSA [28, 30]. Moreover, without PSA testing, most men would only be diagnosed when they become symptomatic, when the disease is often too far advanced to cure. Therefore, the current EAU guidelines recommend opportunistic screening to the well-informed man [31]. Furthermore, there is an urgent need for the development of novel biomarkers.

#### 2.1.1. Opportunistic Screening

Despite the EAU guidelines' recommendation on opportunistic PSA screening, the question remains which patients would benefit the most? Rephrased, we could ask ourselves which men are at an elevated risk of PCa? Since epidemiological studies have suggested a role of genetics in PCa development, it seems tempting to speculate that genetic variations could be of interest in predicting patients' risk of PCa in clinical practice [3]. Using this, clinicians could determine which patients would benefit from PSA screening or in which patient group they should have a low threshold of performing prostate biopsies. Currently, these risk associations, detected by GWAS studies, are of limited clinical utility because the risk prediction is based on comparing groups of people. This allows for patient stratification into “risk groups,” having an x-fold greater PCa risk relative to the population average. However, for the clinician, it is of greater interest to be able to calculate a patient's absolute risk to develop PCa at a certain given point based on an individual's information.

Evaluating the efficiency of detecting PCa using a model based on family history, age, and genetic variation, Zheng et al. suggested comparable efficiency in detecting PCa when compared to the predictive power of PSA level cutoff of 4.1 ng/mL (see Table 1 for an overview of all SNPs cited in this paper) [32]. At first sight, this would seem irrelevant, since the efficiency of PSA testing itself is low. However, the economic burden of genetic testing could potentially be much lower, since this should only be determined once, whereas PSA levels fluctuate over time and often require multiple testing. The combined predictive performance of PSA plus genetic testing on PCa diagnostics unfortunately did not improve diagnostics once age, PSA level, and family history were known [32, 33].

Indeed, it was argued that SNPs are not good at discriminating cases from controls but might be of better use in identifying men at high risk of PCa [34]. In light of this, Xu et al. developed a prediction model of absolute risk for PCa at a specific age based on the sum of 14 SNPs and family history [35]. Using this model, one could identify a small subset (0.5–1%) of individuals at very high risk (41% and 52% absolute risk in a US and Swedish population) of developing PCa between 55 and 74 years of age. Sun et al. studied the performance of three sets of PCa risk SNPs in predicting PCa, showing that they are efficient at discriminating men who have a considerably elevated risk for PCa (a two- and threefold increase when compared to the population median risk) [36].

These risk predictions seem to have the highest impact in young patients with a family history of PCa [37, 38]. This seems logical, since one is born with a certain inheritable subset of SNPs, which do not change throughout one's life. Therefore, it could be expected that their effects would present in the earlier stages of life. Macinnis et al. developed a risk prediction algorithm for familial PCa, using 26 common variants, predicting the cumulative PCa risk depending on family history (from incidental PCa to highly burdened PCa families) and the number of SNPs (expressed in percentile of a SNP profile) [38].

Generalized, the future role of SNP genotyping in PCa screening seems to lie in detecting men at high risk of (aggressive) disease. Men with a higher likelihood of (aggressive) PCa may choose to begin PCa screening at an earlier age and/or more frequently. They may also pursue preventative measures, including diet/lifestyle intervention and chemoprevention.

It has been estimated that, when compared to age-specific screening, personalized screening would result in 16% fewer men being eligible for screening at a cost of 3% fewer screen-detectable cases [39]. Importantly, SNPs can be determined with high accuracy, at low cost and at any age, which make them attractive candidates to predict an individual's risk for PCa.

#### 2.1.2. SNPs in Interpreting (Novel) Biomarkers Levels

SNP genotyping can result in a risk prediction, that is, estimating the likelihood of developing PCa. Currently, they have no role as true diagnostic markers. However, as Klein et al. have already stated, there might be other clinical uses for SNPs [40]. Hypothetically, they could be used in combination with PSA levels, increasing its predictive role [41, 42].

Furthermore, a profound knowledge on SNPs influencing novel biomarker levels would be of great interest, since this could play a crucial role in the interpretation of novel biomarkers. Fundamental research has already identified multiple SNPs playing a role in expression and/or function of hK2, *β*-MSP, TMPRSS2, and so forth [43–45], which could potentially have an important impact on their interpretation.

Recent evidence has suggested that PSA levels are subject to genetic variation as well, explaining 40–45% of variability in PSA levels in the general population [46]. This variability plays an important role in the low sensitivity and specificity of PSA testing, because of which there is no generalizable threshold at which men should undergo prostate biopsies.

Attempting to explain this variability, Gudmundsson et al. detected six loci associated with PSA levels, of which four had a combined relative effect on PSA level variation [47]. Other groups have validated this work. One group suggested that genetic correction could influence the risk of PCa per unit increased/decreased PSA [48]. Another group suggested that genetic correction could alter the number of men with an abnormal PSA (based on general biopsy thresholds), preventing up to 15 to 20% of prostatic biopsies, in this way reducing complications and costs and improving quality of life [49].

With the exponentially increasing interest and development of novel biomarkers in PCa, we should keep in mind that SNPs can clearly alter biomarker levels, which is crucial for correct interpretation. If these SNPs are not taken into account, we could foresee similar obstacles as we are seeing now with PSA testing.

### 2.2. Localized Disease

In treating localized PCa, defined as N0 M0 disease [31], there are multiple viable treatment options, each with its indications and contraindications. Based on clinical stage of the disease, age, and comorbidities, clinicians decide to enroll a patient in an active surveillance program or to start active treatment.

#### 2.2.1. Active Surveillance

Active surveillance (defined as close monitoring of PSA levels combined with periodic imaging and repeat biopsies) is currently the golden standard for treating PCa with the lowest risk of cancer progression (cT1-2a, PSA < 10 ng/mL, biopsy Gleason score <6 (at least 10 cores), <2 positive biopsies, minimal biopsy core involvement (<50% cancer per biopsy)).

Although active surveillance has shown to be a viable option with excellent survival rates, reported conversion rates to active treatment range from 14% to 41%. This delayed treatment, however, has no effect on survival rates [50]. Biomarker research in the “active surveillance group” should therefore mainly focus on detecting high-risk disease with high specificity, avoiding under- and overtreatment and making prevention and early intervention possible. This topic has already been discussed in the paragraph “early detection” (see Section 2.1).

#### 2.2.2. Active Therapy

As it has become clear that, in patients with low-risk disease, active therapy can be delayed based on active surveillance protocols, it is clear as well that therapy is required in patients with intermediate and especially in patients with high-risk disease, as defined by the EAU guidelines.

In light of this, there are two large retrospective studies, determining 15-year mortality rates in men with PCa treated with noncurative intent. Firstly, Rider et al. found that PCa mortality is low in all men diagnosed with localized low- and intermediate-risk PCa. However, death rates are much higher in all men with localized high-risk disease with a 31% mortality rate [51]. Similar results were published by Akre et al., who have shown that in men with locally advanced PCa with a Gleason score of 7–10, where the PCa is managed with noncurative intent, PCa-specific mortality rates range between 41 and 64% [52].

Reported incidence rates of high-risk PCa vary between 17% and 31%, depending on how the disease was defined [53]. After primary treatment, patients with high-risk PCa have a higher risk of disease recurrence and progression, requiring multimodality treatment [31]. Currently, the two most established treatment options with curative intent are surgery and radiotherapy, each with its obstacles.

*Radical Prostatectomy.* In treating patients with intermediate- and high-risk PCa, radical prostatectomy can be curative for some patients, whilst others need a multidisciplinary approach. For clinicians, it is therefore of great interest to be able to define a patient's risk for persistence or relapse of disease before they start treatment. Based on these predictions, clinicians could decide to start with or withhold from adjuvant therapy, leading to a more personalized medicine.

At this moment, these predictions are based on nomograms, integrating preoperative clinical parameters, optimizing their prognostic value [54–56]. However, current clinical parameters still lack sufficient accuracy. Therefore, there is an enormous interest in developing novel biomarkers to optimize pretreatment risk stratification [57]. Regarding this, SNPs are especially interesting, since their use avoids PCa heterogeneity in a single specimen, limiting its pathological parameter accuracy [58, 59]. Again, SNPs are not age-dependent and only need to be determined once, reducing patient burden and costs.

Multiple groups have identified SNPs that might be associated with higher rates of biochemical recurrence and/or shorter periods of biochemical-recurrence-free survival after radical prostatectomy. These polymorphisms are located in genes involved in steroid hormone biotransformation [60–62], immune response [63], Wnt [64] and IGF1 [65] signaling pathways, and other genes associated with oncogenesis [66–68]. However, conflicting results have already been reported by Bachman et al., who found that the AA genotype of the 938 BCL2 promotor polymorphism was an independent prognostic marker of relapse-free and overall survival in a Caucasian patient group [69]. This contrasted with results published by Hirata et al., who found the CC variant of the same promotor to be predictive for biochemical recurrence in a Japanese population [70].

Despite the numerous investigations on the role of SNPs in predicting biochemical recurrence after radical prostatectomy, only a few have been developed into a clinically applicable model, integrating clinicopathological data and genetic information [71, 72].

*Radiotherapy.* External beam radiotherapy (EBRT) is a second treatment option with curative intent for localized PCa. Currently, there is no solid evidence suggesting superiority of surgery or radiotherapy over the other. With the development of new techniques (IMRT, tomotherapy, and so forth) with escalating radiation doses, there has been an increase in patients being treated with EBRT for localized PCa.

Still, toxicities in neighboring normal tissues remain the major limiting factor for delivering optimal tumoricidal doses [73]. Normal-tissue radiation sensitivity mainly depends on treatment-related factors, which are defined as the total irradiation dose, the fractionated regimen, the total treatment time, and the irradiated volume. However, even for similar or identical treatment protocols, the extent of side effects shows substantial variation. This interindividual variation can be explained by patient-related factors [74].

Since patients with higher rates of side effects show no specific phenotypic trait, this suggests that subclinical genetic variations could explain these interindividual differences. Therefore, there has been an increasing interest in identifying the role of genetic variation and SNPs on treatment efficacy and normal-tissue radiosensitivity, termed “radiogenomics.” Detecting these genetic variations could lead to the identification of subgroups of patients at risk for developing severe radiation-induced toxicity [75].

Based on a mechanistic understanding of the radiation pathogenesis, there has been a major interest in understanding the role of genetic variation in genes involved in DNA damage sensing (e.g., ATM), fibrogenesis (e.g., TGFB1), oxidative stress (e.g., SOD1), and major DNA repair pathways (e.g., XRCC1, XRCC3, ERCC2, and MLH1), showing conflicting results [73, 76–83]. Comparable studies have been performed in patients treated with brachytherapy [84–88].

These results are of great interest, since long-term genitourinary, sexual, and gastrointestinal quality of life are major issues guiding decision making with respect to curative management of PCa. In 2012, Barnett et al. aimed to prospectively validate SNPs which were at that time reported to be associated with radiation toxicity in a population of 637 patients treated with radical prostate radiotherapy. Despite previous evidence, none of the 92 investigated SNPs were associated with late normal tissue toxicity [89].

### 2.3. Metastatic Prostate Cancer

Since androgens have a pivotal role in the development of PCa, the androgen receptor is the main target of systemic therapy for PCa. Androgen deprivation therapy (ADT) is the mainstay of treatment for patients with metastatic PCa, of which chemical or surgical castration is the first-line treatment. Because of comparable efficacy between chemical and surgical castration, the latter generally has been replaced by chemical castration [90–92].

#### 2.3.1. Genetic Polymorphisms and ADT

Despite the efficiency of hormonal therapy in metastatic disease, eventually every patient will relapse, developing castration-resistant PCa (CRPC) [93]. Clinicians use well-studied clinicopathologic parameters (PSA kinetics and Gleason Score and so forth) to predict which patients will not respond well to ADT and which patients have poor prognosis [94–96]. Still, these parameters are insufficient for prediction, which is suggested by the recommendation of the EAU guidelines that LHRH agonists should be continued, even in a castration-resistant state [31]. In light of this, genetic markers could be an attractive way to improve risk stratification, predicting which patients will respond less to ADT and have poor prognosis, warranting closer follow-up.

Ross et al. underlined the importance of pharmacogenomics on an individuals' response to ADT [97]. They associated three SNPs located in/close to CYP19A1 (encodes for aromatase, a key enzyme that converts testosterone to estrogen in men), HSD3B1 (associated with PCa susceptibility) [98], and HSD17B4 (overexpression associated with higher Gleason grade) [99]. These SNPs were significantly associated with time to progression, having an additive effect when combined.

Later on, SNPs in multiple other loci have shown to be correlated with earlier relapse in patients treated with ADT. Currently known loci of interest are situated in the EGF gene [100] (known to activate several prooncogenic signaling pathways), in two androgen transporter genes (SLCO2B1 and SLCO1B3) [101] and in the TGF*β*R2 gene. The latter codes for a receptor involved in TGF*β* signaling pathway, playing a role in carcinogenesis and tumor progression [102]. In contrast to these, some SNPs associated with time to progression under ADT, are located in genes of which the function is still unknown [103].

Moreover, a Taiwanese group developed a DNA library of 601 men with “advanced prostate cancer” treated with ADT, in which they detected 5 SNPs that were correlated with progression and 14 SNPs correlated with PCa-specific mortality under ADT [104–107]. Bao et al. detected four SNPs within miRNAs and miRNA target sites that were associated with disease progression [104]. Furthermore, Huang et al. systematically investigated 55 and 49 common SNPs in androgen- and estrogen-receptor-binding sites, after which they withheld one SNP (located in BNC2) which is correlated with progression and 5 SNPs (located in ARRDC3, FLT1, SKAP1, BNC2, and TACC2), which are correlated with PCa-specific mortality [105, 106]. Finally, Huang et al. associated a SNP in the BMP5 and IRS2 gene with survival [107]. The latter encodes a member of a family of intracellular signaling adaptor proteins that coordinate numerous biologically key extracellular signals within the cell, including insulin-like growth factor 1 (IGF1), of which the genotype seems to be correlated with survival in metastatic PCa as well [108]. This only shows how complex and interwoven the clinical effects of genetic variation can be.

Although these results are very interesting, it should be noted that the investigated patient group is very heterogeneous. In this population with “advanced PCa,” tumor characteristics show that 33% of patients have a Gleason score ranging from 2 to 6 and 31.7% of patients have T1/T2 tumors. Furthermore, the setting in which ADT was given was very heterogeneous, ranging from ADT in neoadjuvant setting to ADT for biochemical failure after radical prostatectomy. This heterogeneity limits the interpretation of genetic variation in clinical situations such as these.

Hypothetically, there are multiple potential clinical benefits of SNP genotyping with respect to ADT therapy. Firstly, it could play a prognostic role in identifying patients at high risk of therapeutic failure. This could help identify a subset of patients who may benefit from a more aggressive initial treatment strategy than ADT alone, including combinations with novel drugs [103]. Secondly, polymorphisms with functional implications on enzyme activity could be targeted with novel therapeutics, improving ADT efficacy [97].

#### 2.3.2. Genetic Polymorphisms in the Castrate-Resistant PCa

In the castrate-resistant setting, taxane-based chemotherapy (docetaxel) has been the only treatment option for many years, based on two multicenter phase III randomized clinical trials, showing a moderate increase in overall survival [109, 110]. Over the last few years, numerous novel therapeutics have been developed in this setting. However, equally many questions regarding the optimal treatment regimen remain. Clinical evidence showing superiority to one treatment option over the other is severely lacking, keeping clinicians in the dark on the optimal treatment.

When treatment with docetaxel is started, there is a high variability in the clinical response [111]. Therefore, it would be of great interest to be able to predict this response rate before treatment is started. Based on this, the clinician could decide to withhold from docetaxel as first-line treatment and choose another treatment option.

Genetic variation in the CYP1B1 gene has shown to predict outcome in CRPC patients receiving first-line docetaxel. Pastina et al. showed that patients, carrying the CYP1B1 4326 GG genotype, had significantly shorter overall survival rates when compared to patients carrying CYP1B1 4326 GC or CC. Even after correcting for other risk factors (e.g., demographics, pathological, and biochemical characteristics), this genotype remained an independent predictive parameter of risk of death. This suggests that the 4326GG genotype might be a good pharmacogenetic marker of lower prevalence of response to docetaxel in CRPC patients [112]. It is suggested that its role probably lies in the effect it has on the levels of 4-hydroxyestradiol, which is the major CYP1B1 metabolite. The metabolite interferes with the chemotherapy-induced microtubule stabilization and structurally alters docetaxel [113]. Another gene of interest is the ABCB1 gene, which is responsible for a large portion of the systemic efflux of docetaxel. Within this gene, a combination of ABCB1 1236, 2677, and 3435 genotypes seems to be correlated with survival in CRPC patients receiving docetaxel and time to developing neuropathy in patients receiving a combination of docetaxel and thalidomide. The latter is probably due to cumulative effects on toxicity [114].

With the growing number of new treatment options, these results are very interesting for future clinical use. Using these, clinicians could individualize treatment regimens based on a patient's genotype. Similarly, the role of SNPs in predicting therapeutic efficacy of novel therapeutic agents like abiraterone and enzalutamide awaits to be assessed.

## 3. Conclusions

Since the definition of the human genome, the basis for genetic variations that can lead to individual risks for diseases has become more and more clear. Genome-wide association studies (GWAS) have defined groups of SNPs which partially predict increased risk for PCa. As suggested in this review, SNPs have a great potential in predicting patients' risk for PCa and/or therapy response, which could have an important impact in every day clinic.

Although many authors have suggested that genetic information can improve risk prediction and therefore be useful in clinical practice, there are several studies showing contradicting results, limiting their current clinical use. These contradicting results could be explained by multiple reasons. First, studies performed on small, heterogeneous populations might result in high rates of false positive and negative data. Secondly, most conducted studies are based on SNPs which have been correlated with PCa in GWAS studies. Since PCa phenotypes are probably determined by a spectrum of genetic variation (ranging from highly penetrant to low penetrant variations), with possible interdependencies of SNPs, GWAS studies are probably not sufficient to develop a full understanding of these variations in PCa.

Throughout this review, it has become clear that some challenges still remain for translational research on the role of SNPs in PCa. Firstly, clinical studies on SNPs should be performed in well-powered studies, which could give more conclusive results. Secondly, the important challenge for further basic research is to identify the causative SNPs within each linkage equilibrium. Hopefully, these SNPs will not only function as predictors but also give clues to important pathways in PCa development, which could be therapeutic targets.

It will only be after the enrichment of GWAS data by detailed SNP mapping and functional SNP testing that the most relevant SNPs can be analyzed in clinical research. In the future, we expect them to become critical to interpret individualized PCa risk, interindividual biomarker variation, and therapeutic response.

## Figures and Tables

**Table 1:** Listing all the SNPs being discussed in the referred papers.

First author, year [ref.]	Gene(s)/loci investigated	*N*	Endpoint	Significant SNPs	Conclusion
Xu (2009) [35]	8q24, 17q12, 3p12, 7p15, 7q21, 9q33, 10q11, 11q13, 17q24, 22q13, Xp11	4674, 2329	PCa risk prediction	—	A risk prediction model, based on the number of risk alleles of 14 SNPs and family history, can predict a patients' absolute PCa risk.

Sun (2011) [36]	—	4621	PCa risk prediction	rs16901979, rs6983267, rs1447295, rs4430796, rs1855962, rs2660753, rs10486567, rs10993994, rs10896449, rs5945619, rs1465618, rs721048, rs12621278, rs10934853, rs17021918, rs7679673, rs9364554, rs2928679, rs1512268, rs16902094, rs920861, rs4962416, rs7127900, rs12418451, rs8102476, rs2735839, rs5759167	Genetic risk prediction models are interesting to identify a subset of high-risk men at early, curable stage

Salinas (2009) [33]	17q12, 17q24.3, 8q24	2574	PCa risk prediction	rs4430796, rs1859962, rs6983561, rs6983267, rs1447295	Genotyping for five SNPs plus family history is associated with a significant elevation in risk for prostate cancer. They do not improve prediction models for assessing who is at risk of getting or dying from the disease

Lindström (2012) [37]	EHBP1, THADA, ITGA6, EEFSEC, PDLIM5, TET2, SLC22A3, JAZF1, LMTK2, NKX3-1, SLC25A37, CPNE3, CNGB3, MSMB, CTBP2, TCF2, KLK3, BIK, NUDT11	15161	PCa risk prediction	rs721048, rs1465618, rs12621278, rs2660753, rs4857841, rs17021918, rs12500426, rs7679673, rs9364554, rs10486567, rs6465657, rs6465657, rs1512268, rs2928679, rs4961199, rs1016343, rs7841060, rs16901979, rs620861, rs6983267, rs1447295, rs4242382, rs7837688, rs16902094, rs1571801, rs10993994, rs4962416, rs7127900, rs12418451, rs7931342, rs10896449, rs11649743, rs4430796, rs7501939, rs1859962, rs266849, rs2735839, rs5759167, rs5945572, rs5945619	Incorporating genetic information and family history in prostate cancer risk models can be useful for identifying younger men that might benefit from prostate-specific antigen screening

Macinnis (2011) [38]	—	2885	PCa risk prediction	rs721048, rs1465618, rs12621278, rs2660753, rs17021918, rs12500426, rs7679673, rs9364554, rs10486567, rs6465657, rs10505483, rs6983267, rs1447295, rs2928679, rs1512268, rs10086908, rs620861, rs10993994, rs4962416, rs7931342, rs7127900, rs4430796, rs1859962, rs2735839, rs5759167, rs5945619	The authors developed a risk prediction algorithm for familial prostate cancer, taking into account genotyping of 26 SNPs and family history. The algorithm can be used on pedigrees of an arbitrary size or structure

Zheng (2009) [32]	3p12, 7q15, 7q21, 8q24, 9q33, 10q11, 10q13, 17q12, 17q24.3, Xp11	4674	PCa risk prediction	rs2660753, rs10486567, rs6465657, rs16901979, rs6983267, rs1447295, rs1571801, rs10993994, rs10896449, rs4430796, rs1859962, rs5945619C	The predictive performance for prostate cancer using these genetic variants, family history, and age is similar to that of PSA levels

Loeb (2009) [48]	—	1806	Personalized PSA testing	rs10993994, rs2735839, rs2659056	Genotype influences the risk of prostate cancer per unit increase in prostate-specific antigen. Combined use could improve prostate specific antigen test performance

Helfand (2013) [49]	—	964	Personalized PSA testing	rs2736098, rs10788160, rs11067228, rs17632542	Genotyping can be used to adjust a man's measured prostate-specific antigen concentration and potentially delay or prevent unnecessary prostate biopsies

Klein (2012) [40]	JAZF1, MYC, MSMB, NCOA4, IGF2, INS, TH, TPCN2, MYEOV, HNF1B, DPF1, PPP1R14A, SPINT2, KLK3, TTLL1, BIK, NUDT11	3772	PCa risk prediction	rs10486567, rs11228565, rs17632542, rs5759167	Prostate cancer risk prediction with SNPs alone is less accurate than with PSA at baseline, with no benefit from combining SNPs with PSA

Nam (2009) [41]	17q12, 17q24.3, 8q24, ERG, HOGG1-326, KLK2, TNF, 9p22, HPC1, ETV1	3004	Early detection	rs1447295, rs1859962, rs1800629, rs2348763	When incorporated into a nomogram, genotype status contributed more significantly than PSA. The positive predictive value of the PSA test ranged from 42% to 94% depending on the number of variant genotypes carried

Aly (2011) [42]	THADA, EHBP1, ITGA6, EEFSEC, PDLIM5, FLJ20032, SLC22A3, JAZF1, LMTK2, NKX3-1, MSMB, CTBP2, HNF1B, PPP1R14A, KLK3, TNRC6B, BIK, NUDT11, 8q24.21, 11q15.5, 11q13.2, 17q24.3	5241	PCa risk prediction	rs721048, rs12621278, rs7679673, rs10086908, rs1016343, rs13252298, rs6983561, rs16901979, rs16902094, rs6983267, rs1447295, rs10993994, rs7127900, rs10896449, rs11649743, rs4430796, rs1859962, rs8102476, rs2735839, rs5759167, rs5945619	Using a genetic risk score, implemented in a risk-prediction model, there was a 22.7% reduction in biopsies at a cost of missing a PCa diagnosis in 3% of patients characterized as having an aggressive disease

Hirata (2009) [70]	P53, p21, MDM2, PTEN, GNAS1, bcl2	167	BCR after RP	rs2279115	Bcl2 promotor region −938 C/C genotype carriers more frequently show biochemical recurrence than −938 C/A + A/A carriers

Perez (2010) [58]	EGFR	212	BCR after RP	rs8844019	Statistically significant association between the SP and prostate biochemical recurrence after radical prostatectomy

Morote (2010) [71]	KLK2, SULT1A1, TLR4	703	BCR after RP	rs198977, rs9282861, rs11536889	Predicting biochemical recurrence after radical prostatectomy based on clinicopathological data can be significantly improved by including patient genetic information

Audet-Walsh (2011) [61]	SRD5A1, SRD5A2	846	BCR after RP	rs2208532, rs12470143, rs523349, rs4952197, rs518673, rs12470143	Multiple SRD5A1 and SRD5A2 variations are associated with increased/decreased rates of BCR after RP

Audet-Walsh (2012) [60]	HSD17B1, HSD17B2, HSD17B3, HSD17B4, HSD17B5, HSD17B12	526	BCR after RP	rs1364287, rs8059915, rs2955162, rs4243229, rs1119933, rs9934209, rs7201637, rs10739847, rs2257157, rs1810711, rs11037662, rs7928523, rs12800235, rs10838151	Twelve SNPs distributed across HSD17B2, HSD17B3, and HSD17B12 were associated with increased risk of BCR in localized PCa after RP

Jaboin (2011) [67]	MMP7	212	BCR after RP	rs10895304	The A/G genotype is predictive of decreased recurrence-free survival in patients with clinically localized prostate cancer

Wang (2009) [68]	PCGF2 (MEL-18)	124	BCR after RP	rs708692	Patients with the G/G genotype have a significantly higher rate of BCR after RP.

Bachmann (2011) [69]	bcl2	290	BCR after RP	rs2279115	The −938 A/A genotype carriers more frequently show biochemical recurrence than −938 C/A + C/C carriers

Huang (2010) [64]	CTNNB1, APC	307	BCR after RP	rs3846716	There is a potential prognostic role of the GA/AA genotype of the SNP on BCR after RP.

Chang (2013) [65]	IGF1, IGF1R	320	BCR after RP	rs2946834, rs2016347	A genetic interaction between IGF1 rs2946834 and IGF1R rs2016347 is associated with BCR after RP.

Borque (2013) [72]	KLK3, KLK2, SULT1A1, BGLAP	670	BCR after RP	rs2569733, rs198977, rs9282861, rs1800247	A nomogram, including SNPs and clinicopathological factors, improves the preoperative prediction of early BCR after RP

Langsenlehner (2011) [73]	XRCC1	603	RT toxicity	rs25489	The XRCC1 Arg280His polymorphism may be protective against the development of high-grade late toxicity after radiotherapy.

Damaraju (2006) [77]	BRCA1, BRCA2, ESR1, XRCC1, XRCC2, XRCC3, NBN, RAD51, RAD2-52, LIG4, ATM, BCL2, TGFB1, MSH6, ERCC2, XPF, NR3C1, CYP1A1, CYP2C9, CYP2C19, CYP3A5, CYP2D6, CYP11B2, CYP17A1	83	RT toxicity	rs1805386, rs1052555, rs1800716	SNPs in LIG4, ERC22, and CYP2D6 are putative markers to predict individuals at risk for complications arising from radiation therapy

De Langhe (2013) [78]	TGF*β*1	322	RT toxicity	rs1800469, rs1982073	Radical prostatectomy, the presence of pretreatment nocturia symptoms, and the variant alleles of TGF*β*1 −509 C > T and codon 10 T > C are identified as factors involved in the development of acute radiation-induced nocturia when treated with IMRT

Fachal (2012) [79]	ATM, ERCC2, LIG4, MLH1, XRCC3	698	RT toxicity	rs1799794	The SNP and the mean dose received by the rectum are associated with the development of gastrointestinal toxicity after 3D-CRT.

Fachal (2012) [80]	TGF*β*1	413	RT toxicity	None	Neither of the investigated SNPs or haplotypes were found to be associated with the risk of late toxicity.

Popanda (2009) [81]	XRCC1, APEX1, hOGG1, XRCC2, XRCC3, NBN, XPA, ERCC1, XPC, TP53, P21, MDM2	405	RT toxicity	rs25487, rs861539	The XRCC1 Arg399Gln polymorphism is associated with an increase in risk for heterozygous individuals and for Gln carriers. For XRCC3 Thr241Met, the Met variant increases the risk in Met carriers

Suga (2008) [82]	SART1, ID3, EPDR1, PAH, XRCC6	197	RT toxicity	rs2276015, rs2742946, rs1376264, rs1126758, rs2267437	Two-stage AUC-ROC curve reached a maximum of 0.86 (training set) in predicting late genitourinary morbidity

Cesaretti (2005) [85]	ATM	37	RT toxicity (Brachy)	—	There is a strong association between sequence variants in the ATM gene and erectile dysfunction/rectal bleeding

Cesaretti (2007) [84]	ATM	108	RT toxicity (Brachy)	—	The possession of SNPs in the ATM gene is associated with the development of radiation-induced proctitis after brachytherapy

Peters (2008) [86]	TGF*β*1	141	RT toxicity (Brachy)	rs1982073, rs1800469, rs1800471	Presence of certain TGF*β*1 genotypes is associated with the development of both erectile dysfunction and late rectal bleeding in patients treated with radiotherapy.

Pugh (2009) [87]	ATM, BRCA1, ERCC2, H2AFX, LIG4, MDC1, MRE11A, RAD50	41	RT toxicity (Brachy)	rs28986317	The high toxicity group is enriched for at least one LIG4 SNP. One SNP in MDC1 is associated with increased radiosensitivity.

Burri (2008) [88]	SOD2, XRCC1, XRCC3	135	RT toxicity	rs25489, rs4880, rs861539	A XRCC1 SNP is associated with erectile dysfunction. A combination of a SNP in SOD2 and XRCC3 is associated with late rectal bleeding

Barnett (2012) [89]	ABCA1, ALAD, APEX1, ATM, BAX, CD44, CDKN1A, DCLRE1C, EPDR1, ERCC2, ERCC4, GSTA1, GSTP1, HIF1A, IL12RB2, LIG3, LIG4, MED2L2, MAP3K7, MAT1A, MLH1, MPO, MRE11A, MSH2, NEIL3, NFE2L2, NOS3, PAH, PRKDC, PTTG1, RAD17, RAD21, RAD9A, REV3L, SART1, SH3GL1, SOD2, TGFB1, TGFB3, TP53, XPC, XRCC1, XRCC3, XRCC5, XRCC6	637	RT toxicity	None	None of the previously reported associations were confirmed by this study, after adjustment for multiple comparisons. The *P* value distribution of the SNPs tested against overall toxicity score was not different from that expected by chance

Ross (2008) [97]	AKR1C1, AKR1C2, AKR1C3, AR, CYP11A1, CYP11B1, CYP17A1, CYP19A1, CYP21A2, CYP3A4, DHRS9, HSD17B3, HSD17B4, HSD3B1, HSD3B2, MAOA, SRD5A1, SRD5A2, SREBF2, UGT2B15	529	ADT efficacy	rs1870050, rs1856888, rs7737181	Three polymorphisms in separate genes are significantly associated with time to progression during ADT.

Teixeira (2008) [100]	EGF	275	ADT efficacy	rs4444903	EGF functional polymorphism may contribute to earlier relapse in ABT patients, supporting the involvement of EGF as an alternative pathway in hormone-resistant prostatic tumors

Yang (2011) [101]	SLCO2B1, SLCO1B3	538	ADT efficacy	rs12422149, rs1789693, rs1077858	Three SNPs in SLCO2B1 were associated with time to progression (TTP) on ADT. Patients carrying both SLCO2B1 and SLCO1B3 genotypes, which import androgens more efficiently, exhibited a median 2-year shorter TTP on ADT

Teixeira (2013) [102]	TGFBR2	1765	ADT efficacy	—	TGFBR2-875GG homozygous patients have an increased risk of an early relapse after ADT. Combining clinicopathological and genetic information resulted in an increased capacity to predict the risk of ADT failure

Kohli (2012) [103]	TRMT11, HSD17B12, PRMT3, WBSCR22, CYP3A4, PRMT2, SULT2B1, SRD5A1, AKR1D1, UGT2A1, SULT1E, HSD3B1, UGT2A3, UGT2B11, UGT2B28, CYP19A1, PRMT7, METTL2B, HSD17B3, LCMT1, UGT2B7, SRD5A2, CYP11B2, CARM1, METTL6, HSD17B1, HEMK1, CYP11B1, ESR1, UGT2B10, SERPINE1, PRMT6, HSD11B1, THBS1, SULT2A1, UGT2B4, PRMT5, PRMT8, HSD3B2, UGT1A4, ARSE, UGT1A8, UGT1A5, UGT1A10, ESR2, LCMT2, UGT1A9, AR, UGT1A6, UGT1A7, AKR1C4, STS, HSD17B8, ARSD, HSD17B2, HSD17B7, UGT1A1, UGT1A3,	304	ADT efficacy	rs1268121, rs6900796	TRMT11 showed the strongest association with time to ADT failure, with two of 4 TRMT 11 tagSNPs associated with time to ADT failure.

Bao (2011) [104]	KIF3C, CDON, ETS1, IFI30, has-mir-423, PALLD, ACSL1, GABRA1, SYT9, ZDHHC7, MTRR	601	ADT efficacy	rs6728684, rs3737336, rs1045747, rs1071738, rs998754, rs4351800	KIF3C rs6728684, CDON rs3737336, and IFI30 rs1045747 genotypes remained as significant predictors for disease progression in multivariate models that included clinicopathologic predictors. A greater number of unfavorable genotypes were associated with a shorter time to progression and worse prostate cancer-specific survival during ADT.

Huang (2012) [106]	SPRED2, GNPDA2, BNC2, ZNF521, ZNF507, ALPK1, SKAP2, TACC2, SKAP1, KLHL14, NR4A2, FBXO32, AATF	601	ADT efficacy	rs16934641, rs3763763, rs2051778, rs3763763	Genetic variants in BNC2, TACC2, and ALPK1 are associated with clinical outcomes after ADT, with a cumulative effect on ACM following ADT of combinations of genotypes across the two loci of interest.

Huang (2012) [105]	ACTN2, NR2F1, ARRDC3, XRCC6BP1, FLT1, PSMD7, SKAP1, FBXO32, FLRT3	601	ADT efficacy	rs2939244, rs9508016, rs6504145, rs7830622, rs9508016	Genetic variants in ARRDC3, FLT1, and SKAP1 are significant predictors for PCSM and genetic variants in FBXO32 and FLT1 remained significant predictors for ACM. There was a strong combined genotype effect on PCSM and ACM.

Huang (2012) [107]	BMP5, NCOR2, IRS2, MAP2K6, RXRA, ERG, BMPR1A	601	ADT efficacy	rs4862396, rs3734444, rs7986346	Genetic variants in CASP3, BMP5, and IRS2 are associated with ACM. Genetic variation in BMP5 and IRS2 is significantly related to PCSM. Patients carrying a greater number of unfavorable genotypes at the loci of interest have a shorter time to ACM and PCSM during ADT.

Tsuchiya (2013) [108]	IGF-1	251	Metastatic PCa outcome	—	When the sum of the risk genetic factors in each LD block was considered, patients with all the risk factors had significantly shorter cancer-specific survival than those with 0–2 risk factors.

Pastina (2010) [112]	CYP1B1	60	Docetaxel response	rs1056836	The polymorphism is a possible predictive marker of response and clinical outcome to docetaxel in CRPC patients.

Sissung (2008) [113]	CYP1B1	52	Docetaxel response	rs1056836	Individuals carrying two copies of the polymorphic variant have a poor prognosis after docetaxel-based therapies compared with individuals carrying at least one copy of the allele.

Sissung (2008) [114]	ABCB1	73	Docetaxel response	—	Docetaxel-induced neuropathy, neutropenia grade, and overall survival could be linked to ABCB1 allelic variants (diplotypes).

Listing all the studies being discussed. From left to right: author (ref), genes/loci tested, number of patients included in the cohort, general endpoint of the study, significant SNPs, and conclusions.
